# Increased risk of bradycardia in vigorous infants receiving early as compared to delayed cord clamping at birth

**DOI:** 10.1038/s41372-022-01593-1

**Published:** 2022-12-31

**Authors:** Ashish KC, So Yeon Joyce Kong, Solveig Haukås Haaland, Joar Eilevstjønn, Helge Myklebust, Ram Chandra Bastola, Thomas Ragnar Wood, Susan Niermeyer, Sara Berkelhamer

**Affiliations:** 1 Department of Women’s and Children’s Health, Uppsala University, Uppsala, Sweden; 2 Laerdal Medical, Stavanger, Norway; 3 Pokhara Academy of Health Sciences, Pokhara, Nepal; 4 Center on Human Development and Disability, University of Washington, Seattle, WA USA; 5 University of Colorado School of Medicine, Colorado School of Public Health, Aurora, CO USA; 6 Department of Pediatrics, University of Washington, Seattle, WA USA

**Keywords:** Circulation, Epidemiology

## Abstract

**Objective:**

To compare HR pattern of vigorous newborns during the first 180 s with early (≤60 s, ECC) or delayed (>60 s, DCC) cord clamping.

**Study design:**

Observational study including dry-electrode ECG monitoring of 610 vaginally-born singleton term and late-preterm (≥34 weeks) who were vigorous after birth.

**Results:**

198 received ECC while 412 received DCC with median cord clamping at 37 s and 94 s. Median HR remained stable from 30 to 180 s with DCC (172 and 170 bpm respectively) but increased with ECC (169 and 184 bpm). The proportion with bradycardia was higher among ECC than DCC at 30 s and fell faster in the DCC through 60 s. After adjusting for factors affecting timing of cord clamping, ECC had significant risk of bradycardia compared to DCC (aRR 1.51; 95% CI; 1.01–2.26).

**Conclusion:**

Early heart instability and higher risk of bradycardia with ECC as compared to DCC supports the recommended clinical practice of DCC.

## Introduction

During the first minutes after birth, two key physiological events—aeration of the newborn lungs and umbilical cord clamping—impact the successful transition of neonates from the intra-uterine to extra-uterine environment [1–3]. Crying at birth generates large trans-pulmonary pressure differences and increases blood oxygen content through pulmonary aeration. As a result, pulmonary vascular resistance drops, resulting in increased pulmonary blood flow and transition of flow across the ductus arteriosus from the fetal pattern of right-to-left to dominant left-to-right [4]. Umbilical cord clamping at birth plays a critical role in this transition with respect to hemodynamics, as umbilical venous return supplies preload for the left ventricle through the patent foramen ovale during fetal life. Clamping the umbilical cord limits cardiac venous return in the absence of adequate ventilation and pulmonary blood flow [5]. As a result, cardiac output falls precipitously and may remain compromised, resulting in bradycardia. Heart rate (HR) is used as a critical indicator of the need for resuscitation [6]. As per the International Liaison Committee on Resuscitation (ILCOR) guidelines and Neonatal Resuscitation Program a HR below 100 beats per minute (bpm) in an infant who is not breathing well or crying despite stimulation indicates the need for positive-pressure ventilation (PPV) [7].

Generally, a crying infant is assumed to have a heart rate >100, and reflex bradycardia is uncommon with cord clamping in vigorous, crying infants [8]. However, studies which have evaluated normative HRs have provided inconsistent findings regarding rates of bradycardia in these well infants.

Dawson et al. reported HR centile curves in the first 10 minutes after birth among 466 neonates who did not require intervention [9]. Early cord clamping (<1 min) was the standard of care at the time this study was performed. A pulse oximeter placed on infants’ right wrist collected and stored HR data every 2 seconds. At 1 minute, 50% of vigorous neonates had a HR less than 100 bpm and 17% of neonates had a HR less than 60 bpm, raising questions about using HR < 100 bpm as a threshold requiring intervention with PPV. Several studies have shown that pulse oximetry reports lower HRs than electrocardiography (ECG) and exhibits a time lag to detection [10–13]. In the delivery room, ECG is more effective than pulse oximetry in providing timely and accurate information on HR and is recommended when resuscitation is needed [11].

Bjorland et al. demonstrated that using a wireless dry-electrode ECG monitor with display (NeoBeat, Laerdal Global Health, Stavanger, Norway) the HR centile graphs were re-evaluated in 898 term vigorous, vaginally born neonates with delayed cord clamping (after 1 minute) [14]. A dry-electrode ECG is significantly faster to place and acquires HR more efficiently than pulse oximetry [15]. This study showed that HRs <100 bpm after 1 minute were uncommon in healthy newborns after delayed cord clamping. The differences in HR at 1 minute as reported by Dawson and Bjorland may derive not only from different measurement methods, but also from the timing of cord clamping, reflecting the critical physiologic changes and the potential benefit of ongoing placental transfusion even in a vigorous infants during the first few minutes after birth [9, 14].

We hypothesized that timing of cord clamping (early or delayed) may impact normative HR data in vigorous infants. We hypothesized that early cord clamping would be associated with more bradycardia even in vigorous infants. Both Helping Babies Breathe (HBB) and the Neonatal Resuscitation Program (NRP) recommend that the initial steps of resuscitation can be performed with the umbilical cord intact if the infant is vigorous. As providers in our study were trained in HBB, we defined delayed cord clamping as >60 seconds as recommended with the HBB algorithm. With data from directly observed deliveries including the timing of cord clamping and respiratory status synchronized with continuous HR data obtained by dry electrode, we aimed to assess HR pattern in the first 180 seconds after birth among vigorous infants who received early cord clamping (ECC) as compared to delayed cord clamping (DCC).

## Materials/subjects and methods

### Design

The study was conducted with a single-center, prospective, observational design.

### Setting

The study was conducted at Pokhara Academy of Health Sciences, Pokhara, Nepal from May to December 2020. The hospital had 6500 annual births and is the referral hospital of the province. A nurse-midwife attends all births with on-call support from a general medical doctor, pediatrician and obstetrician when needed.

REFINE, a heart-rate-guided neonatal resuscitation protocol, was implemented by providing training in Helping Babies Breathe (HBB) supplemented by a half-day orientation on the use of NeoBeat at all births [16]. With this curriculum, providers were taught to provide delayed cord clamping if an infant was vigorous, which was defined as maintaining the cord intact for at least 60 seconds in HBB. Within REFINE, periodic meetings were facilitated by the quality improvement team to discuss progress on implementation of heart-rate-guided neonatal resuscitation care using a Plan-Do-Study-Act (PDSA) process [17].

### Participants

All women admitted to the labor room with a singleton pregnancy at gestational age 34 weeks or more were eligible for this study. Eligible women who agreed to participate provided written consent for enrollment. Infants born by caesarean section were not included as observation during childbirth in the operation theatre was not feasible. All newborns who were not vigorous at birth or required respiratory support (bag mask ventilation) were excluded from this study. Vigorous newborns were defined as those who cried immediately (within approximately 15 seconds) after birth and required no intervention beyond thorough drying immediately after birth.

### Data collection and management

Study research nurses were present in the labor and delivery room to collect data using mobile device with a purpose-built Liveborn application to record real-time observations of newborn care in the first few minutes after birth as previously described [18]. A research nurse attending the delivery noted time of birth, time of cord clamping, time of infant cry, and start and stop of resuscitation interventions such as stimulation, suctioning, and ventilation by annotating the events in the Liveborn application. After delivery the neonate was thoroughly dried by the health worker and a research nurse applied NeoBeat on the upper abdomen. NeoBeat incorporates two dry electrodes into a semi-circular carrier with an integrated digital display that is easily visible to the provider (Supplementary Fig. 1). Continuous HR data was recorded by was recorded by Neobeat, live streamed to the Liveborn application and synchronized with activities recorded in the application. Movement or handling of the baby could temporarily prevent detection or cause interference with HR.

Vigorous newborns were positioned on the mother’s chest after NeoBeat was placed. Drying of newborns was done on mother’s chest. Cord clamping was performed at the health worker’s discretion, although training on HBB recommended cord clamping after 1 minute and the majority of providers had received recent training. Demographics, obstetric history and obstetric complications during labour (Supplementary Table 1) and neonatal characteristics were collected from medical records [19]. The Liveborn application automatically integrated heart rate with any intervention provided after time of birth. A single unique ID was provided for each participant to link the Liveborn app and demographic/medical history data for final data analysis.

### Data analysis

For the data analysis, we excluded a participant’s data when there was an indication that the birth time registered in Liveborn application was not correct, i.e. if NeoBeat recording started before “baby born” was pressed in the Liveborn application. Other exclusion criteria included too little (<30 s), too late (>120 s after birth) or poor signal quality data. We categorized the participants into two different groups based on the timing of umbilical cord clamping, defined as early cord clamping (ECC) at 60 seconds or less and delayed cord clamping (DCC) at 61 seconds or more. The obstetric characteristics of ECC and DCC were analyzed including mean maternal age, parity, induction of labor, gestation, and obstetric complications during labor. The neonatal characteristics analyzed included gestational age in weeks, birth weight in grams, gender, Apgar score at 1 and 5 minutes, time of first HR in seconds and immediate drying after birth. Means were compared using independent *t*-test, medians by Mann-Whitney *U* test and proportions via Pearson’s Chi-square test.

We analyzed the continuous HR data for the ECC and DCC groups from 10 until 180 seconds after birth using centile curves at 3rd, 10th, 25th, 50th, 75th, 90th and 97th. The centile curves were smoothed using piecewise cubic spline fitting. Median HRs at 12 serial time points (10, 30, 45, 60, 75, 90, 105, 120, 135, 150, 165 and 180 s) were compared using Mann Whitney *U* test with *post hoc* Bonferroni correction of p-values to adjust for multiple repeated measures (*p* = 0.05/12 = *p* ≤ 0.004 for significance).

Proportion of bradycardic infants with 95% CI in the ECC and DCC groups was graphically presented using generalized additive models (GAM). The relative risk of ever being bradycardic (HR < 100 bpm) after cord clamping was determined by cord clamping group after birth using generalized linear model regression with binomial distribution and log link function. Gestational age in weeks, birthweight in grams, presence of bradycardia at time of cord clamp, and obstetric complications during labor were included as covariates. Results are presented as the crude relative risk (cRR) and adjusted relative risk (aRR), with 95% confidence intervals (CI). Analyses were performed using R version 44.1.2 (Vienna, Austria) and Matlab R2021a (Mathworks Inc, Natick, MA).

## Results

### Study population

**Fig. 1:**
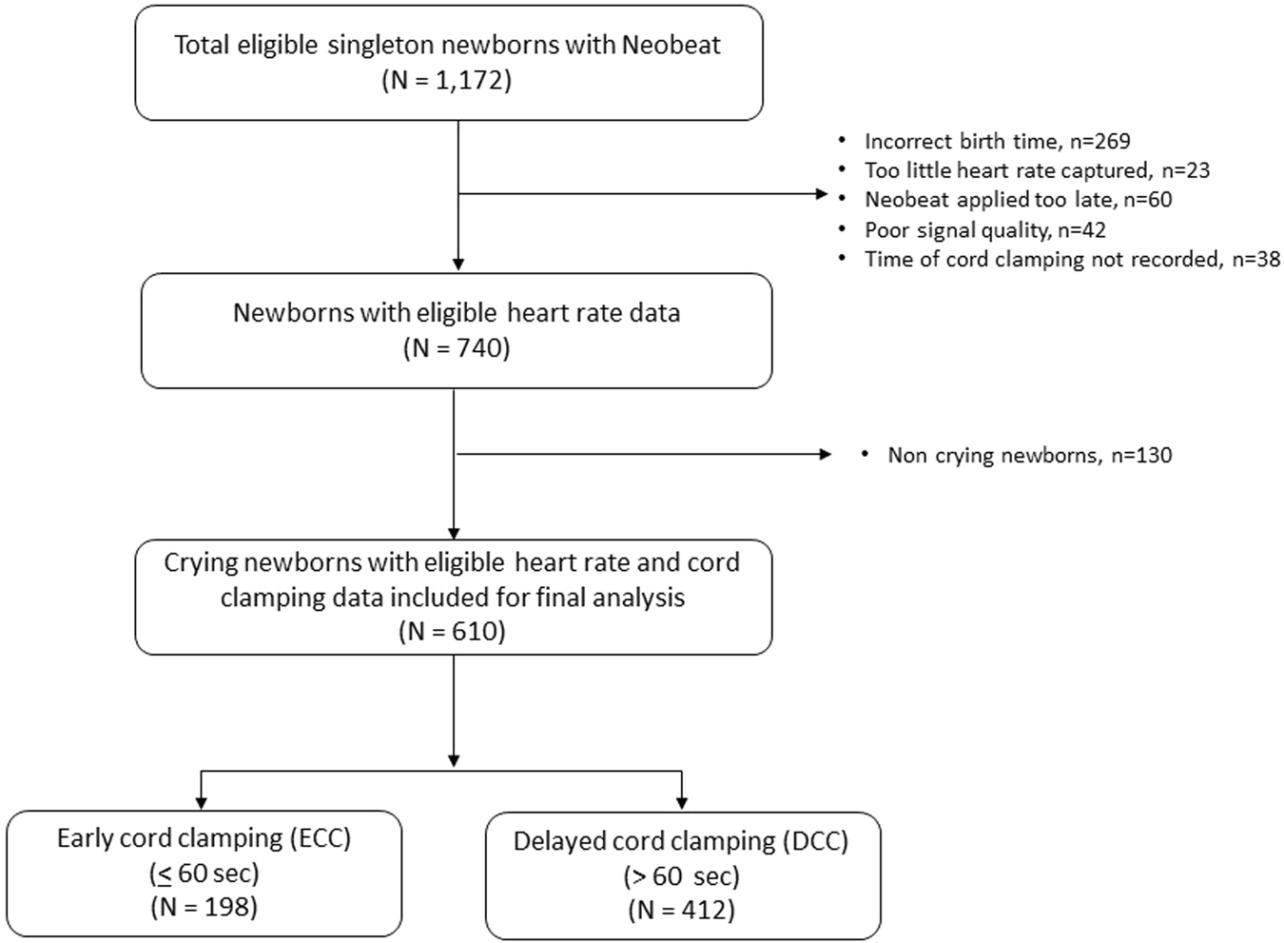
Study population. Flow diagram of study population.

During the study period, NeoBeat was applied on 1172 neonates. Of them, 269 were excluded due to incorrect birth time or duplicate case ID, 23 were excluded due to insufficient HR data captured (<30 s), 60 were excluded due late application of NeoBeat to the upper abdomen (>120 s after birth), 42 were excluded due to poor signal quality, and 38 cases were excluded due to not having a cord clamp time registered in Liveborn. Among the remaining 740 neonates, 610 were vigorous neonates and included in the final analysis. Of the neonates included, 198 had cord clamping at 60 seconds or less, and 412 had cord clamping after 60 seconds (Fig. 1).

### Patient characteristics

Comparison of obstetric and neonatal characteristics of the ECC and DCC cohorts identified that the cohorts were largely similar, with the exception that the ECC group had a higher rate of obstructed labor (18.7% vs 10.7%; *p* = 0.01). Comparable maternal age, parity, birthweight, gestational age, rates of induction, gender distribution and Apgar scores were observed. (Table 1).

**Table 1:** Maternal and newborn characteristics in ECC and DCC cohorts.

	Early cord clamping (ECC) (*N* = 198)	Delayed cord clamping (DCC) (*N* = 412)	*p*-value
Mother’s age, years (mean ± SD)	24.1 ± 4.3	24.2 ± 4.6	0.56
Parity, Primiparous (%, *n*)	52.1% (110)	52.4% (217)	0.54
Birth weight, grams (mean ± SD)	3080.8 ± 424.8	3030.7 ± 412.8	0.79
Gestational age, weeks (mean ± SD)	39.1 ± 1.6	39.2 ± 1.4	0.37
Obstetric complications at labor (%, *n*)	26.8% (53)	16.7% (69)	0.01
Fever during labor	1.0% (2)	0.0% (0)	0.11
Multiple pregnancies	0.5% (1)	0.0% (0)	0.33
Maternal pre-existing medical complication	0.0% (0)	0.5% (2)	0.46
Obstructed labour	18.7% (37)	10.7% (44)	0.01
Oligohydramnios	4.0% (8)	2.4% (10)	0.20
Hypertensive disorder	0.0% (0)	0.5% (2)	0.46
Premature rupture of membrane	2.0% (4)	2.4% (10)	0.50
Maternal diabetes	0.5% (1)	0.0% (0)	0.33
Congenital fetal malformation	0.0% (0)	0.2% (1)	0.68
Induction of labour (%, *n*)	16.1% (34)	17.4% (72)	0.76
Sex of Infant, Female (%, *n*)	42.7% (90)	42.5% (176)	0.64
Apgar score at 1 minute (mean ± SD)	7 ± 0.6	7 ± 0.3	0.21
Apgar score at 5 minutes (mean ± SD)	8 ± 0.5	8 ± 0.2	0.43

Statistical comparison by independent *t*-test and Pearson’s chi-square test.

*SD* standard deviation, *N* number, *HR* heart rate.

The proportion of neonates dried was comparable in the ECC and DCC groups (99.0% vs 98.5%, *p* = 0.49). The median times (quartiles) to cord clamping were 37 (25, 48) seconds among ECC and 94 (75, 121) seconds among the DCC (*p* < 0.0001). There was no significant difference in median time (quartiles) to HR in ECC as compared to DCC (17.0 [12.0, 23.0] vs 17.0 [13.0, 23.5], *p* = 0.68). The proportion of infants with bradycardia any time before cord clamping was comparable between the groups (9.1% vs 9.5%, *p* = 0.88). However, in 2% of infants in the ECC group, the first HR was obtained after cord clamping, while HR was obtained before clamping for all infants in the DCC group. The median HR [bpm] at the time of cord clamping was similar between the groups (169.0 [145.2, 183.8] vs 168.0 [153.0, 182.0], *p* = 0.63) (Table 2).

**Table 2:** Time to cord clamping and heart rate between ECC and DCC cohorts.

Statistic	ECC (*n* = 198)	DCC (*n* = 412)	*p*-value
Time to cord clamp [s], median (p25, p75)	37.0 (26.0, 48.0)	94.0 (75.0, 121.0)	<0.01
Time to first HR [s], median (p25, p75)	17.0 (12.0, 23.0)	17.0 (13.0, 23.5)	0.68
First (detected) HR [bpm], median (p25, p75)	159.5 (126.0, 177.0)	165.5 (141.5, 180.0)	0.09
First HR detected before cord clamp	84.8% (168)	99.8% (411)	<0.01
Bradycardic (<100 bpm) first HR	11.1% (22)	9.5% (39)	0.53
Bradycardic and first HR before cord clamp	9.1% (18)	9.5% (39)	0.88
Bradycardic and cord clamp before first HR	2.0% (4)	0.0% (0)	<0.01
	*n* = *167*	*n* = *370*	
HR at cord clamp [bpm], median (p25, p75)	169.0 (145.2, 183.8)	168.0 (153.0, 182.0)	0.63
HR change from cord clamp to 15 s after cord clamp [bpm], median (p25, p75)	0.0 (−6.0, 9.8)	1.0 (−4.0, 7.0)	0.40

*ECC* early cord clamping, *DCC* delayed cord clamping, *s* second, *p25* 25th percentile, *p75* 75th percentile, *HR* heart rate, *bpm* beats per minute.

### Heart rate centiles

**Fig. 2:**
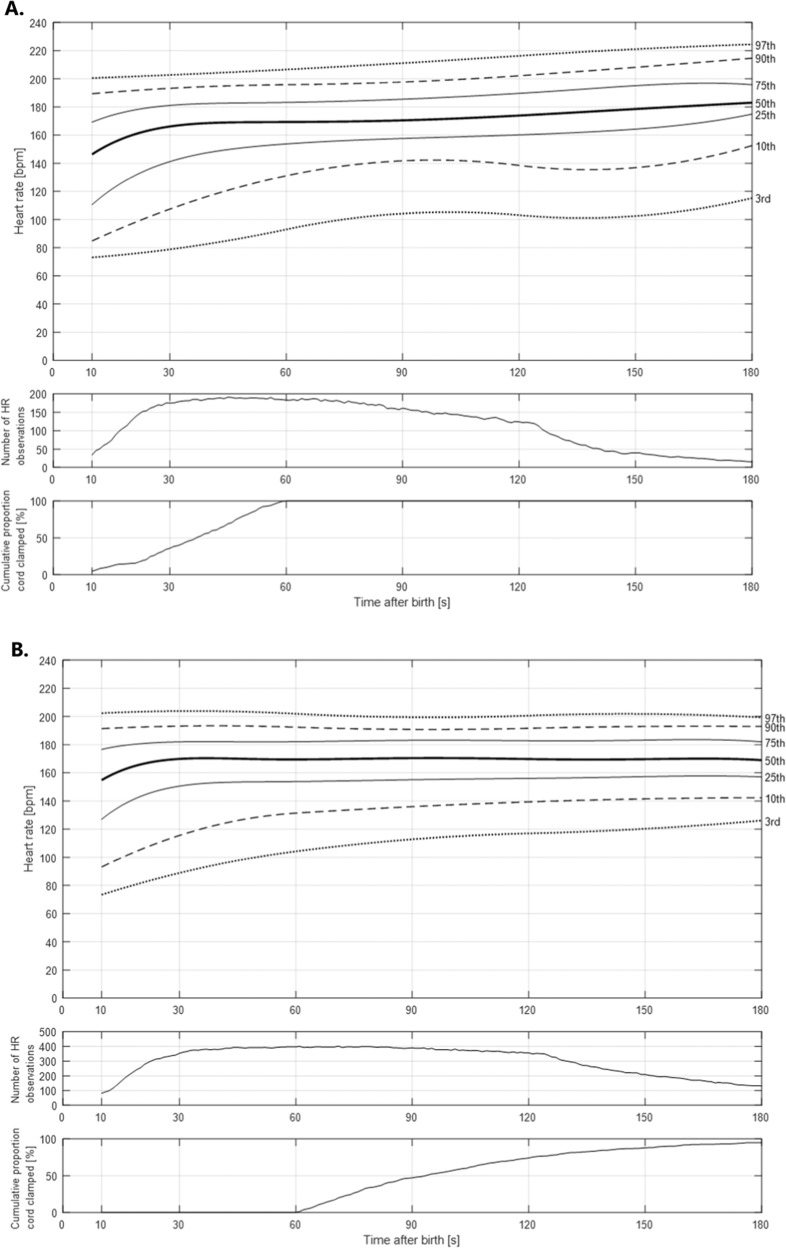
Smoothed heart rate percentiles in vigorous infants. **A** Smoothed heart rate percentiles in vigorous infants with early cord clamping. **B** Smoothed heart rate percentiles in vigorous infants with delayed cord clamping.

The 3rd, 10th, 25th, 50th, 75th, 90th and 97th centile smoothed HRs in ECC and DCC groups are displayed in Fig. 2A, B (numerical values are listed in Supplementary Tables 2 and 3). The 3rd centile HR in the ECC cohort remained below 100 bpm to 60 seconds and 3rd centile HR in the DCC cohort remained below 100 bpm to 45 seconds. There were no significant differences in median HRs from 10 to 180 seconds after birth between ECC and DCC groups at the prespecified time intervals. However, median HRs [bpm] remained stable from 30 to 180 seconds with DCC [172 (152, 183) at 30 s and 170 (156, 184) at 180 s] whereas gradual increase was noted with ECC [169 (143, 183) at 30 s and 184 (173, 198) at 180 s] (Supplementary Table 4).

### Risk of bradycardia following cord clamping

Compared to the DCC group with ECC, the crude risk of being bradycardic did not defer (cRR 0.91; 95% CI; 0.61–1.36; *p* < 0.64). After adjustment for factors which influenced timing of cord clamping including gestational age, birthweight, presence of bradycardia at time of cord clamp, and obstructed labor, ECC group had a significantly greater risk of bradycardia after cord clamping compared to the DCC group (aRR 1.51; 95% CI; 1.01–2.26; *p* = 0.047) (Table 3). In sensitivity analysis without obstructed labour in both groups, in the fully adjusted model at the time of cord clamping, RR for ever being bradycardic after cord clamping in the ECC group was 1.57 higher relative to the DCC group (aRR 1.57; 95% CI; 1.05–2.35; *p* = 0.027) (Supplementary Table 5 and Supplementary Fig. 2). In additional sensitivity analysis without all obstetric complication in both groups, in the fully adjusted model at the time of cord clamping, adjusted Odds ratio for ever being bradycardic after cord clamping in the ECC group was 1.83 higher relative to the DCC group not statistically significant (aOR 1.83; 95% CI; 0.95–3.53; *p* = 0.07) (Supplementary Table 6 and Supplementary Fig. 3).

**Table 3:** Relative risk (RR) for ever being bradycardic after cord clamping in the ECC group relative to the DCC group with 95% confidence interval (CI).

Predictor	RR (95% CI, *p*-value)	
	Crude	Adjusted
Early Cord Clamping	0.91 (0.61–1.36; 0.64)	1.51 (1.01–2.26; 0.047)
Birthweight (per kg)	-	1.33 (0.87–2.04; 0.19)
Gestational Age (per week)	-	0.99 (0.87–1.13; 0.92)
Obstructed Labor	-	0.79 (0.45–1.39; 0.41)
Bradycardia at cord clamp	-	5.09 (3.46–7.48; <0.001)

Fully-adjusted model includes birth weight, gestational age, obstructed labor, and presence of bradycardia at the time of cord clamping.

**Fig. 3:**
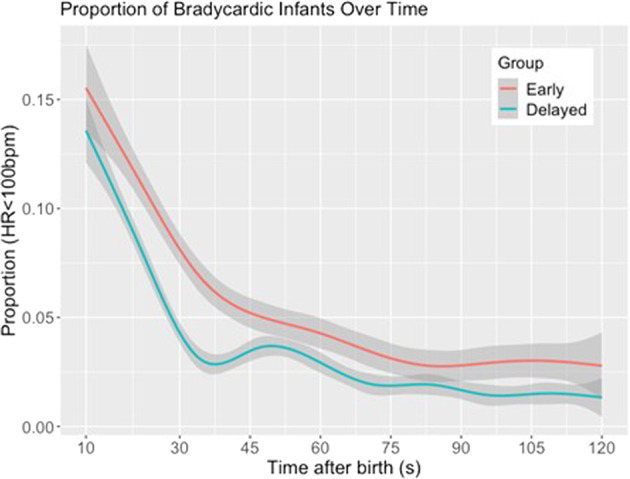
Proportion of vigorous neonates with heart rate less than 100 bpm with ECC and DCC. GAM curves with 95%CI displaying the proportion of infants who were bradycardic in the first 2 minutes after birth, stratified by cord clamping time.

The proportion of infants who were bradycardic was similar 10 s after birth in the ECC and DCC groups (8.8% vs 12.3%, *p* = 0.59). Bradycardia was more common in the ECC group at both 30 and 45 s (8.0% vs 3.1%, *p* = 0.01; 6.3% vs 2.8%, *p* = 0.04) then remained similar in groups until 120 seconds. The proportion of babies with bradycardia fell faster in the DCC as compared to ECC group through 60 s (Fig. 3 and Supplementary Table 7).

## Discussion

Our data demonstrates comparable median HRs in vigorous infants who receive ECC and DCC, with more stable HR over the first few minutes in infants who received DCC. While these data are from non-randomized observations, they suggest higher rates of bradycardia in the first few minutes in infants who received ECC adjusting for risk factors for use of ECC. ECC may therein place infants at risk for further deterioration with resuscitative interventions such as airway suctioning. Rapid decline in bradycardia among the cohort of vigorous infants who received DCC further suggests that transplacental transfusion may provide added benefit by improving cardiovenous return and support cardiac output even with adequate lung aeration.

Our study provides an update to data provided by Dawson et al. [9] including a HR of less than 100 bpm at 180 seconds among 7% of vigorous neonates who received early cord clamping. Our data suggests that even with ECC this degree of bradycardia is rare beyond the first minute of life and does not replicate the gradual rise in heart rates observed in the Dawson cohort which was obtained with less accurate and less efficient pulse oximeter [10]. Our results align more closely with Bjorland et al. [14], who also used dry electrode ECG and observed less than 3% of their DCC study population having HR less than 100 at 30 seconds and more rapid stabilization of heart rate over the first few minutes.

Delayed cord clamping, defined as that occurring beyond 1 min after birth, is the recommended standard of care by the World Health Organization worldwide with growing recognition of its benefits on cardiovascular transition and newborn outcomes [20–22]. Prior attempts to prospectively define HR have yielded different results and were done in spontaneously breathing infants not infants who cried after birth. Our previously published randomized controlled study among the spontaneously breathing infants using pulse oximetry identified lower median HRs with DCC as compared to ECC in healthy late preterm and term neonates [23]. In contrast Padilla-Sanchez et al. used pulse oximetry but documented higher HRs with use of DCC [24]. These studies differed in inclusion of near term versus term infants as well as did not include only crying infants, which may have impacted disparate findings.

NeoBeat has been shown to have accuracy comparable to that of a conventional 3-lead ECG monitor in newborns and can be placed within seconds of birth [25]. Our data utilizing this more robust technology more accurately characterizes early heart rate patterns and identified higher adjusted rates of bradycardia with ECC.

### Methodological consideration

There are several limitations to this study. The main limitation is potential selection bias of neonates included in ECC and DCC cohort allocation of into ECC and DCC was not randomized. While our study population was defined as vigorous infants, some neonates who received early cord clamping might have had more subtle clinical indicators leading to an anticipated need for resuscitation. However, the presence of obstetric factors and other demographics which might impact timing of cord clamping were controlled for, we identified an increased risk of bradycardia with ECC. While randomization would be optimal to minimize the selection bias, it provides ethical challenges as DCC is recommended by the WHO for all infants who are vigorous at birth [26, 27]. Despite being trained to delay at least 1-minute, notable variation in timing of cord clamping existed in our dataset with about 1/3 of the infants receiving ECC.

Clinical research staff were used to observe and document the events that occurred following birth, including the time of birth, presence of cry and timing of cord clamping. There is potential for observer bias in classification (vigorous) or miss documentation of events. Data entered manually during a busy resuscitation may have inaccuracies as multiple events need to be documented in a short time period.

Acknowledging these limitations, we would highlight that this pragmatic study provides high-fidelity data on HR pattern immediately following birth in vigorous neonates and impact of timing of cord clamping. While early cord clamping did not impact the median HR in vigorous infants, it resulted in greater HR instability at birth and a higher risk of bradycardia. Further prospective evaluations are indicated in addition to thoughtful review of thresholds for intervention during resuscitation, as our data supports that a HR less than 100 bpm may not represent pathology among vigorous infants. Algorithms for care of the newborn may need to be updated to consider the impact of a more stable transition with the standardization of DCC.

## Disclaimer

The external funding sources had no role in study design, data collection, data analysis, data interpretation, writing of the report or in the decision to submit the paper for publication.

## Supplementary information

Supplementary figure and tables. Increased risk of bradycardia in vigorous infants receiving early as compared to delayed cord clamping at birth

## Data Availability

We have provided as Supplementary File.
