# Supplementary material for: Increased risk of bradycardia in vigorous infants receiving early as compared to delayed cord clamping at birth
Source: J Perinatol. 2022 Dec 31;43(6):709–15. doi: 10.1038/s41372-022-01593-1 (PMC10256607; doi:10.1038/s41372-022-01593-1)
Supplement: Supplementary file 1 — Supplementary figure and tables. Increased risk of bradycardia in vigorous infants receiving early as compared to delayed cord clamping at birth [file 41372_2022_1593_MOESM1_ESM.docx]

**Supplementary figure and tables.** **Increased risk of bradycardia in vigorous infants receiving early as compared to delayed cord clamping at birth**

Ashish KC, So Yeon Joyce Kong, Solveig Haukås Haaland, Joar Eilevstjønn, Helge Myklebust, Ram Chandra Bastola, Thomas Ragnar Wood, Susan Niermeyer, Sara Berkelhamer

Contents

[**Supplementary figure 1.** NeoBeat with visualization of heart rate display on a term infant. 3](#_Toc116467004)

[**Supplementary figure 2**. Proportion of vigorous neonates with heart rate less than 100 bpm by cord clamping status after removing infants with obstructed labor (Sensitivity analysis 1). 4](#_Toc116467005)

[**Supplementary figure 3.** Proportion of vigorous neonates with heart rate less than 100 bpm by cord clamping status after removing all infants with obstetric complications (Sensitivity analysis 2). 5](#_Toc116467006)

[**Supplementary table 1.** Clinical definition of obstetric complication 6](#_Toc116467007)

[**Supplementary table 2.** Smoothed heart rate centiles of early cord clamped crying neonates in the first 3 minutes after birth (corresponding to Figure 2A) 7](#_Toc116467008)

[**Supplementary table 3.** Smoothed heart rate centiles of delayed cord clamped crying neonates in the first 3 minutes after birth (corresponding to Figure 2B) 7](#_Toc116467009)

[**Supplementary table 4.** Comparison of median heart rate (bpm) between early vs delayed cord clamping. P values of < 0.004 were considered significant (p=0.05/12) using individual Mann-Whitney U-tests followed by post hoc Bonferroni correction for multiple comparisons. 8](#_Toc116467010)

[**Supplementary table 5.** Sensitivity analysis - Relative risk (RR) for ever being bradycardic after cord clamping in the DCC group relative to the ECC group with 95% confidence interval (CI) after removing infants with obstructed labor 8](#_Toc116467011)

[**Supplementary table 6**. Sensitivity analysis - odds ratio (OR) for ever being bradycardic after cord clamping in the DCC group relative to the ECC group with 95% confidence interval (CI) after removing all infants with obstetric complications 9](#_Toc116467012)

[**Supplementary Table 7.** Comparison of proportion of vigorous neonates with heart rate less than 100 bpm with ECC and DCC using Fischer’s exact test. 9](#_Toc116467013)

# **Supplementary figure 1.** NeoBeat with visualization of heart rate display on a term infant.


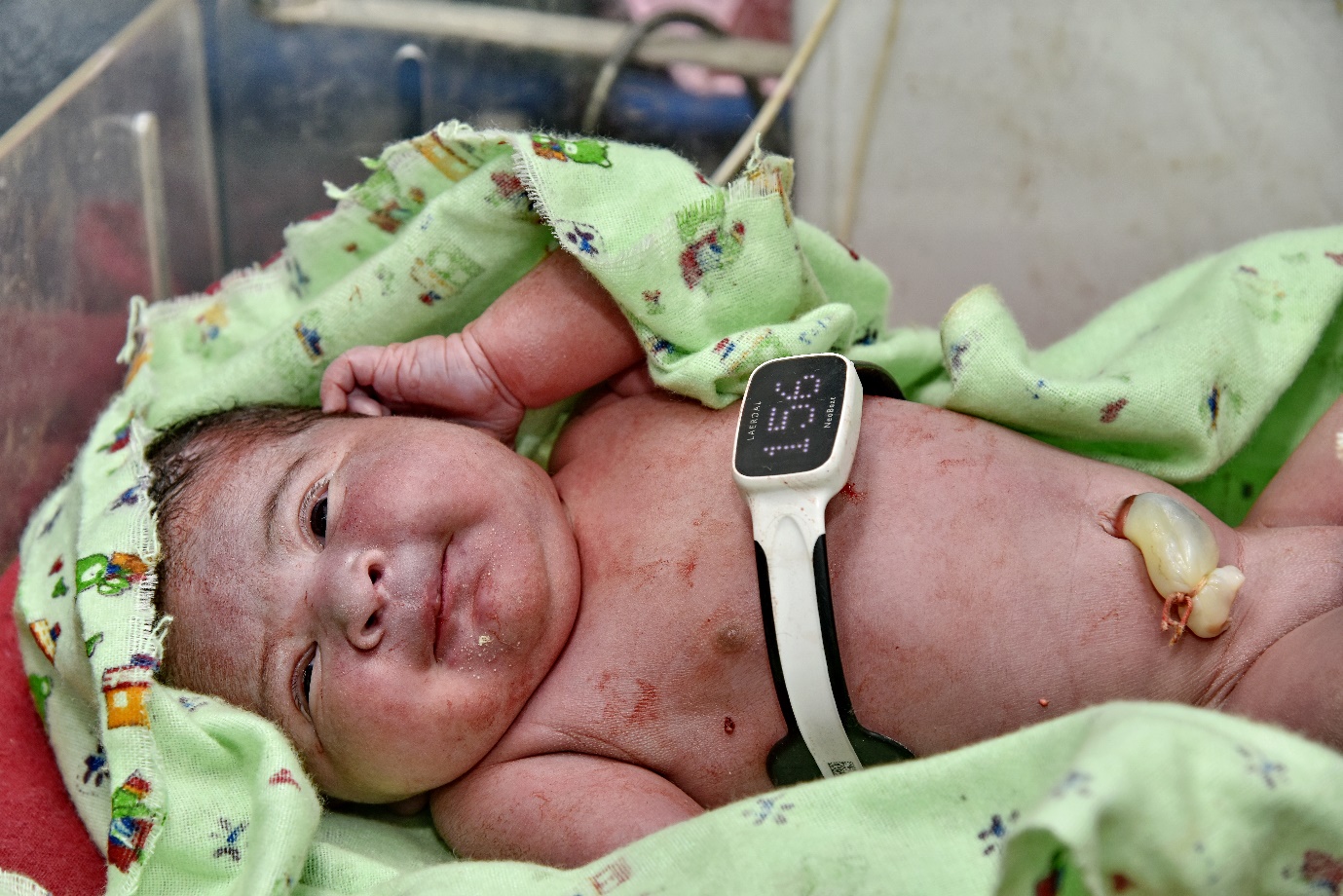


# **Supplementary figure 2**. Proportion of vigorous neonates with heart rate less than 100 bpm by cord clamping status after removing infants with obstructed labor (Sensitivity analysis 1).


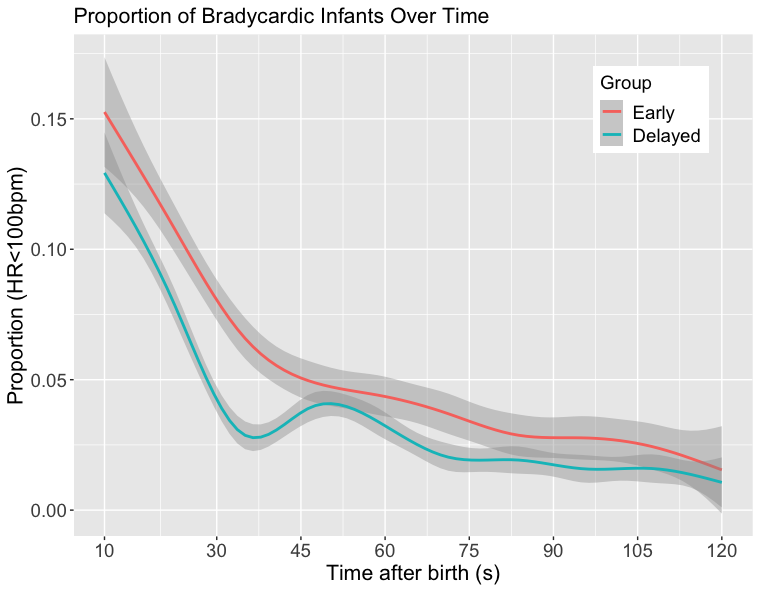


GAM curves with 95%CI displaying the proportion of infants who were bradycardic in the first 2 minutes after birth, stratified by cord clamping time.

# **Supplementary figure 3.** Proportion of vigorous neonates with heart rate less than 100 bpm by cord clamping status after removing all infants with obstetric complications (Sensitivity analysis 2).


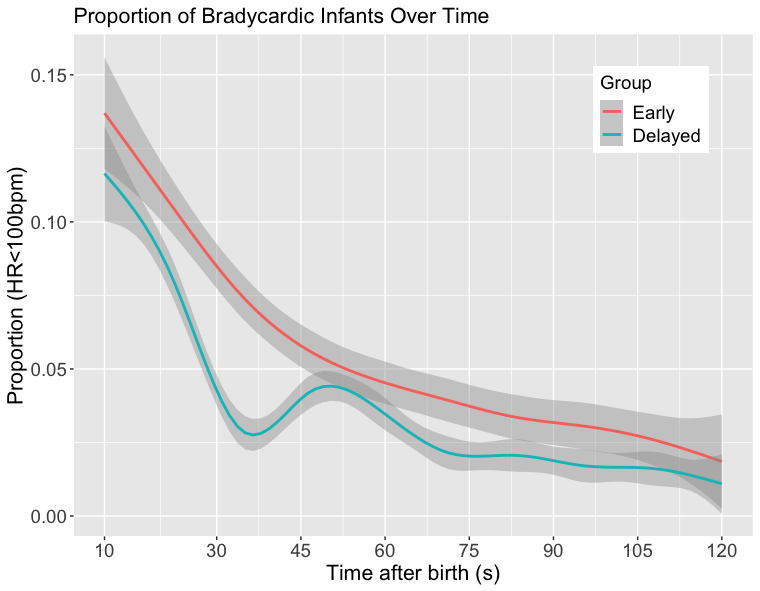


GAM curves with 95%CI displaying the proportion of infants who were bradycardic in the first 2 minutes after birth, stratified by cord clamping time.

# **Supplementary table 1.** Clinical definition of obstetric complication

| Fever during labor | A woman has a fever (temperature of 38°C or more) during labor. |
| --- | --- |
| Multiple pregnancies | If the woman was pregnant with more than one fetus. |
| Abruptio placenta | Abruptio placentae is the detachment of a normally located placenta from the uterus before birth of the baby. |
| Maternal pre-existing medical complication | Any medical maternal condition existing before pregnancy |
| Obstructed labour | Secondary arrest of cervical dilatation and descent of presenting part with large caput, third degree moulding, cervix poorly applied to presenting part, oedematous cervix, ballooning of lower uterine segment, formation of retraction band, or maternal and fetal distress |
| Oligohydraminos | Oligohydramnios refers to the condition that there is inadequate fluid in the amniotic sac of the expectant mother |
| Premature Rupture of Membrane | Prelabor rupture of membranes (PROM) is rupture of the membranes before labor has begun. PROM can occur either when the fetus is immature or preterm (i.e. before 37 weeks)—also called preterm prelabor rupture of membranes (PPROM)—or when the fetus is mature (term). |
| Hypertensive disorder | Occurred when the maternal diastolic blood pressure was 90 mmHg or more for two consecutive readings. |
| Malpresentation | When the fetus presented in any other position than the vertex presentation. |
| Prolonged labor | The cervix is not dilated beyond 4 cm after eight hours of regular contractions. OR, Cervical dilatation is to the right of the alert line on the partograph. OR, the woman has been experiencing labor pains for 12 hours or more  without giving birth (prolonged labor). OR, the cervix is fully dilated and the woman has the urge to push, but  there is no descent. |
| Prolapsed cord | When the umbilical cord was present in the birth canal below the fetal presenting part or the umbilical cord was visible at the vagina following the rupture of membranes. |
| Gestational diabetes | Diabetes that develops in pregnancy |
| Loss of fetal moment | Fetal movements are not felt after 22 weeks of gestation or during labor |
| Prolapsed cord | The umbilical cord lies in the birth canal below the fetal presenting part. The umbilical cord is visible at the vagina following rupture of the  membranes. |
| Fetal distress | Abnormal fetal heart rate (less than 100 or more than  180 beats per minute). Thick meconium-stained amniotic fluid |
| Congenital birth malformation | Major birth defect detected at birth |

# **Supplementary table 2.** Smoothed heart rate centiles of early cord clamped crying neonates in the first 3 minutes after birth (corresponding to Figure 2A)

| Seconds after birth | Heart rate (bpm) centiles | | | | | | |
| --- | --- | --- | --- | --- | --- | --- | --- |
|  | 3^rd^ | 10^th^ | 25^th^ | 50^th^ | 75^th^ | 90^th^ | 97^th^ |
| **10** | 69 | 87 | 111 | 146 | 169 | 187 | 202 |
| **30** | 81 | 107 | 141 | 166 | 181 | 194 | 202 |
| **45** | 85 | 122 | 150 | 169 | 183 | 194 | 205 |
| **60** | 92 | 132 | 154 | 169 | 183 | 195 | 207 |
| **75** | 100 | 137 | 156 | 170 | 184 | 197 | 209 |
| **90** | 105 | 140 | 158 | 171 | 185 | 199 | 211 |
| **105** | 106 | 142 | 159 | 172 | 187 | 200 | 214 |
| **120** | 103 | 141 | 160 | 174 | 190 | 201 | 216 |
| **135** | 101 | 138 | 162 | 176 | 192 | 204 | 219 |
| **150** | 102 | 135 | 164 | 179 | 195 | 209 | 221 |
| **165** | 107 | 139 | 168 | 181 | 197 | 213 | 223 |
| **180** | 115 | 163 | 175 | 183 | 196 | 210 | 224 |

# **Supplementary table 3.** Smoothed heart rate centiles of delayed cord clamped crying neonates in the first 3 minutes after birth (corresponding to Figure 2B)

| Seconds after birth | Heart rate (bpm) centiles | | | | | | |
| --- | --- | --- | --- | --- | --- | --- | --- |
|  | 3^rd^ | 10^th^ | 25^th^ | 50^th^ | 75^th^ | 90^th^ | 97^th^ |
| **10** | 63 | 86 | 127 | 155 | 177 | 190 | 203 |
| **30** | 94 | 119 | 151 | 170 | 182 | 194 | 204 |
| **45** | 97 | 126 | 153 | 170 | 182 | 193 | 203 |
| **60** | 101 | 129 | 154 | 169 | 182 | 192 | 202 |
| **75** | 107 | 133 | 154 | 170 | 183 | 192 | 201 |
| **90** | 113 | 136 | 155 | 170 | 183 | 191 | 200 |
| **105** | 118 | 139 | 156 | 170 | 183 | 191 | 199 |
| **120** | 120 | 140 | 156 | 170 | 183 | 192 | 200 |
| **135** | 119 | 141 | 157 | 169 | 183 | 192 | 202 |
| **150** | 118 | 141 | 157 | 170 | 183 | 193 | 202 |
| **165** | 120 | 141 | 158 | 170 | 184 | 194 | 201 |
| **180** | 134 | 144 | 157 | 169 | 182 | 192 | 199 |

# **Supplementary table 4.** Comparison of median heart rate (bpm) between early vs delayed cord clamping. P values of < 0.004 were considered significant (p=0.05/12) using individual Mann-Whitney U-tests followed by post hoc Bonferroni correction for multiple comparisons.

|  | Early cord clamping | | Delayed cord clamping | |  |
| --- | --- | --- | --- | --- | --- |
| Time | Median (quartiles) | N | Median (quartiles) | N | p-value |
| 10 s | 144 (119, 171) | 34 | 157 (136, 182) | 81 | 0.35 |
| 30 s | 169 (143, 183) | 175 | 172 (152, 183) | 353 | 0.14 |
| 45 s | 168 (151, 183) | 191 | 169 (154, 182) | 393 | 0.62 |
| 60 s | 168 (153, 184) | 183 | 169 (153, 182) | 397 | 0.72 |
| 75 s | 172 (156, 183) | 175 | 170 (156, 183) | 395 | 0.50 |
| 90 s | 171 (157, 184) | 161 | 170 (154, 184) | 390 | 0.15 |
| 105 s | 172 (160, 188) | 142 | 170 (154, 184) | 371 | 0.07 |
| 120 s | 173 (162, 188) | 124 | 169 (156, 183) | 356 | 0.01 |
| 135 s | 178 (159, 193) | 64 | 170 (156, 183) | 270 | 0.02 |
| 150 s | 179 (157, 193) | 40 | 169 (158, 183) | 210 | 0.05 |
| 165 s | 178 (170, 194) | 26 | 170 (158, 184) | 167 | 0.03 |
| 180 s | 184 (173, 198) | 16 | 170 (156, 184) | 132 | 0.01 |

# **Supplementary table 5.** Sensitivity analysis - Relative risk (RR) for ever being bradycardic after cord clamping in the DCC group relative to the ECC group with 95% confidence interval (CI) after removing infants with obstructed labor

|  | **RR (95% CI; p-value)** | | |
| --- | --- | --- | --- |
| **Predictor** | Crude | Adjusted |  |
| Early Cord Clamping | 0.91 (0.60-1.43; 0.72) | **1.57 (1.05-2.35; 0.027)** |  |
| Birthweight (per kg) |  | 1.34 (0.87-2.07; 0.19) |  |
| Gestational Age (per week) |  | 0.98 (0.87-1.12; 0.81) |  |
| Bradycardia at cord clamp |  | **5.05 (3.42-7.47; <0.001)** |  |

(n=522 infants included). Fully adjusted model includes birth weight, gestational age, and presence of bradycardia at the time of cord clamping.

# **Supplementary table 6**. Sensitivity analysis - odds ratio (OR) for ever being bradycardic after cord clamping in the DCC group relative to the ECC group with 95% confidence interval (CI) after removing all infants with obstetric complications

|  | **OR (95% CI; p-value)** | |
| --- | --- | --- |
| **Predictor** | Crude | Adjusted |
| Early Cord Clamping | 1.01 (0.59-1.73; 0.98) | 1.83 (0.95-3.53; 0.07) |
| Birthweight (per kg) | - | 1.06 (0.55-2.05; 0.86) |
| Gestational Age (per week) | - | 1.12 (0.91-1.37; 0.30) |
| Bradycardia at cord clamp | - | **10.5 (4.8-22.9; <0.001)** |

N=482 infants included. Fully adjusted model includes birth weight, gestational age, and presence of bradycardia at the time of cord clamping. N.B. Logistic regression used due to non-convergence of relative risk regression model.

# **Supplementary Table 7.** Comparison of proportion of vigorous neonates with heart rate less than 100 bpm with ECC and DCC using Fischer’s exact test.

| **Statistic** | **ECC (n=198)** | **DCC (n=412)** | **p** |
| --- | --- | --- | --- |
| 10 s | 8.8% (3/34) | 12.3% (10/81) | 0.59 |
| 30 s | 8.0% (14/175) | 3.1% (11/353) | 0.01 |
| 45 s | 6.3% (12/191) | 2.8% (11/393) | 0.04 |
| 60 s | 4.4% (8/183) | 3.0% (12/397) | 0.41 |
| 75 s | 2.9% (5/175) | 2.0% (8/395) | 0.55 |
| 90 s | 2.5% (4/161) | 1.8% (7/390) | 0.74 |
| 105 s | 2.1% (3/142) | 1.3% (5/371) | 0.69 |
| 120 s | 2.4% (3/124) | 1.1% (4/356) | 0.38 |
